# Recent and future declines of a historically widespread pollinator linked to climate, land cover, and pesticides

**DOI:** 10.1073/pnas.2211223120

**Published:** 2023-01-23

**Authors:** William M. Janousek, Margaret R. Douglas, Syd Cannings, Marion A. Clément, Casey M. Delphia, Jeffrey G. Everett, Richard G. Hatfield, Douglas A. Keinath, Jonathan B. Uhuad Koch, Lindsie M. McCabe, John M. Mola, Jane E. Ogilvie, Imtiaz Rangwala, Leif L. Richardson, Ashley T. Rohde, James P. Strange, Lusha M. Tronstad, Tabitha A. Graves

**Affiliations:** ^a^U.S. Geological Survey, Northern Rocky Mountain Science Center, West Glacier, MT 59936; ^b^Department of Environmental Studies and Environmental Science, Dickinson College, Carlisle, PA 17013; ^c^Canadian Wildlife Service, Environment and Climate Change Canada, Whitehorse, YT, Canada Y1A 5X7; ^d^U.S. Fish and Wildlife Service, Cheyenne, WY 82007; ^e^Montana State University, Bozeman, MT 59717; ^f^U.S. Fish and Wildlife Service, Portland, OR 97267; ^g^Xerces Society for Invertebrate Conservation, Portland, OR 97232; ^h^U.S. Department of Agriculture–Agricultural Research Service–Pollinating Insect Biology, Management, Systematics Research Unit, Logan, UT 84341; ^i^U.S. Geological Survey, Fort Collins Science Center, Fort Collins, CO 80526; ^j^Rocky Mountain Biological Laboratory, Crested Butte, CO 81224; ^k^North Central Climate Adaptation Science Center and Cooperative Institute for Research in Environmental Sciences, University of Colorado Boulder, Boulder, CO 80309; ^l^Department of Entomology, The Ohio State University, Columbus, OH 43210; ^m^Wyoming Natural Diversity Database, University of Wyoming, Laramie, WY 82071

**Keywords:** bumble bees, climate change, insect declines, neonicotinoids, biodiversity loss

## Abstract

The acute decline in global biodiversity includes not only the loss of rare species, but also the rapid collapse of common species across many different taxa. The loss of pollinating insects is of particular concern because of the ecological and economic values these species provide. The western bumble bee (*Bombus occidentalis*) was once common in western North America, but this species has become increasingly rare through much of its range. To understand potential mechanisms driving these declines, we used Bayesian occupancy models to investigate the effects of climate and land cover from 1998 to 2020, pesticide use from 2008 to 2014, and projected expected occupancy under three future scenarios. Using 14,457 surveys across 2.8 million km^2^ in the western United States, we found strong negative relationships between increasing temperature and drought on occupancy and identified neonicotinoids as the pesticides of greatest negative influence across our study region. The mean predicted occupancy declined by 57% from 1998 to 2020, ranging from 15 to 83% declines across 16 ecoregions. Even under the most optimistic scenario, we found continued declines in nearly half of the ecoregions by the 2050s and mean declines of 93% under the most severe scenario across all ecoregions. This assessment underscores the tenuous future of *B. occidentalis* and demonstrates the scale of stressors likely contributing to rapid loss of related pollinator species throughout the globe. Scaled-up, international species-monitoring schemes and improved integration of data from formal surveys and community science will substantively improve the understanding of stressors and bumble bee population trends.

## Significance

One dramatic impact of the acute decline in global biodiversity includes losses of once-common species and the potential cascading effects of their absences on ecosystems. Using 23 y of data, 14,457 surveys across 2.8 million km^2 ^in the western United States, we demonstrate negative impacts of increasing temperatures and drought and identify nitroguanidine neonicotinoids as the pesticides most impacting the formerly common pollinator, the western bumble bee (*Bombus occidentalis*). By the 2050s, our most optimistic scenario predicts occupancy declines in almost half of ecoregions; more severe scenarios predict declines in all ecoregions ranging from 51 to 97%. The precipitous decline of this generalist species is a bellwether for loss across many taxa sensitive to environmental changes around the globe.

There are many examples of the loss of an iconic species where the culprit was unmistakable and the outcome was clear, but the reasons why common species decline is often not obvious and likely due to multiple causes. During the 19th century, shotgun blasts across central North America signaled the demise of the passenger pigeon (*Ectopistes migratorius*), once estimated to be 40% of the total bird population in the continent with up to 2 billion individuals (1). Over a century later, we have libraries of laws, policies, and best practices guiding conservation efforts, yet we find ourselves amid a new period of mass extinction (2, 3). Today, species’ declines often stem from indirect effects of human activities such as climate change or environmental degradation via gradual land conversion. Increasingly, common but understudied species are quietly disappearing over short time periods. The global decline of insect pollinators is an example of such a pattern (3–5).

By facilitating reproduction in over 85% of flowering plants (6), pollinators provide a critical ecosystem function, create the foundation of terrestrial food webs, and serve as a linchpin to cascading effects across species (7). Many forbs have coevolved mutualistic relationships with pollinators, with forbs providing nectar or pollen and pollinators increasing forb fecundity (seed set) by orders of magnitude (8). Forbs in turn provide a food resource for other taxa, including species of conservation need and economic interest. For example, greater sage-grouse (*Centrocercus urophasianus*) depends on early spring forbs during brooding (9) and brown bear’s (*Ursus arctos*) seasonal diets can consist largely of flower roots such as sweet vetch (*Hedysarum* spp.) (10). Pollinators also support food webs by serving as food for insectivorous birds and lizards and other arthropods such as dragonflies, ants, and spiders (11). Losing even a single common pollinator species can disrupt the entire pollinator networks (7, 12), with abrupt consequences for the species that directly or indirectly rely on them for food, including humans which benefit from crop pollination services of $1.5 billion annually in the United States alone (13). Because of the integral role of pollinators across ecosystems, especially related to species of concern and human benefit, it is imperative to evaluate the drivers of pollinator declines.

Drastic declines in pollinators are well represented by the story of the western bumble bee (*Bombus occidentalis*) which was once broadly distributed and locally abundant throughout western North America (14). The historical distribution of *B. occidentalis* extended from alpine regions of Arizona and New Mexico to central British Columbia and from the Pacific coastline east to the Black Hills of South Dakota (14, 15). As demand for greenhouse pollination grew in the 1990s, industrial scale rearing and commercialization of *B. occidentalis* grew as well due to the species’ biological and economic attributes (16). Starting in 1996, captive reared *B. occidentalis* populations distributed across North America to greenhouses and open field settings for pollination services experienced increased disease, particularly the fungus *Vairimorpha* (previously *Nosema*) *bombi* (17), providing a likely source of disease to wild colonies. Captive rearing became unsustainable due to disease impacts on managed colonies and was abandoned by the commercial bumble bee industry in the early 2000s (18). The demise of commercial *B. occidentalis* populations coincided with declines observed in wild *B. occidentalis* starting in 1998 in southern Oregon (19), urban San Francisco (20), and British Columbia (21). An independent assessment of *V. bombi* in museum specimens demonstrated increased prevalence of the pathogen in wild *B. occidentalis* starting in 1994 (22), supporting the hypothesis that the continental spread of *V. bombi* via the commercial pollination industry contributed to the wild *B. occidentalis* decline into the early 2000s (5).

In 2015, the U.S. Fish and Wildlife Service (FWS) was petitioned to list *B. occidentalis* as threatened or endangered under the Endangered Species Act (23). Studies attributed declines of *B. occidentalis* populations to *V. bombi*, but also to additional factors including other diseases, habitat destruction, lethal and sublethal effects of pesticides, climate change, competition with nonnative honey bees (*Apis mellifera*), and high vulnerability of the species to negative genetic factors arising from reductions in population size due to haplodiploid reproduction (24, 25). Most information regarding the impacts of these threats has been drawn from localized studies and studies of surrogate bumble bee species. No studies have quantitatively investigated the cumulative effects of changing climate, land cover, and pesticide use. Therefore, the effect of each of these stressors, independently or concurrently, on *B. occidentalis* at range-wide scales remains unclear, subsequently leaving best management directions equally uncertain.

Disentangling the relative contribution of potential drivers to range-wide trends necessitates an extensive investigation using methods that can integrate diverse data sources across the multidecadal time span of declines. Prior research has investigated the relationships between bumble bee species declines and a variety of stressors (24, 25). However, studies of *Bombus* species rarely evaluate multiple types of stressors simultaneously to allow for a more direct comparison of mechanisms of decline while also employing analytical methods that account for the imperfect detection of species during surveys. Failure to account for imperfect detection results in the confounding of the occupancy and detection processes and the degree of bias introduced is unknowable (26). Hierarchical models provide a less biased approach by separating the observation process involving sampling error from the biological processes of interest.

**Fig. 1.**
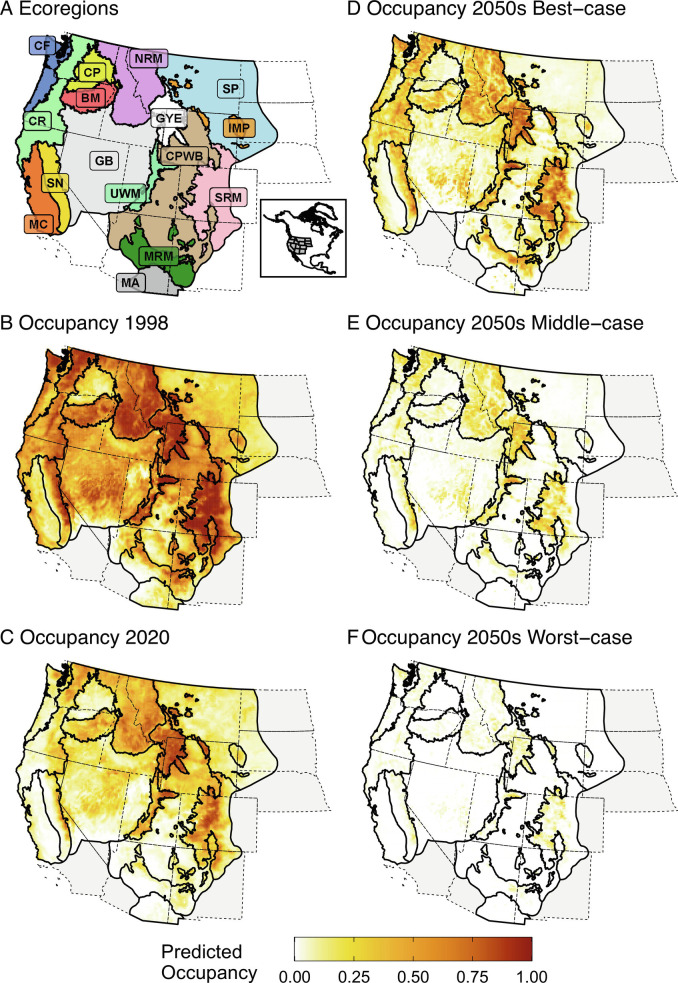
Maps of ecoregions used to assess changes in the occupancy of *B. occidentalis* (*A*), mean predicted occupancy in 1998 and 2020 (*B*, *C*), and projected mean occupancy under three future scenarios (*D*, *E*, *F*) in the western conterminous United States.

Here, we build on previous research and apply robust quantitative methods that account for imperfect detection to test competing mechanisms associated with the decline of this once-widespread North American pollinator. We use hierarchical Bayesian occupancy models to investigate the contributions of climate, land cover, and pesticide use to trends in *B. occidentalis* occupancy across the western conterminous United States. We investigate the effects of climate and land cover on *B. occidentalis* occupancy from 1998 to 2020 and incorporate the effects of pesticides in a subset analysis from 2008 to 2014. We evaluate trends in occupancy across the range of *B. occidentalis* and among ecoregions representing unique geographic areas where populations experience varying intensities of stressors (Fig. 1*A*). In addition to estimating contemporary, spatially explicit trends using preexisting and newly collected bumble bee survey data, we also use a scenario approach to project future occupancy to the mid-century (2050 to 2059). Our unique approach to project future declines blends multiple climate models, emission levels, land cover change forecasts, and potential effects from other continued stressors to generate three future scenarios encompassing a range of plausible outcomes.

## Results

Across the species’ range, the mean predicted occupancy for *B. occidentalis* was 0.48 (95% CRI = 0.40, 0.58) in 1998 and declined to 0.21 (95% CRI = 0.18, 0.25) by 2020 (Fig. 1 *B* and *C*); a mean decline of 57% (95% CRI = 45%, 66%). All ecoregions exhibited declines in *B. occidentalis* from 1998 to 2020, but these declines varied in magnitude from 15% in the Greater Yellowstone Ecosystem to 83% in the Madrean Archipelago ecoregion (Table 1). The largest absolute decline in occupancy from 1998 to 2020 was observed in the Cascade Range ecoregion with a mean reduction of 0.42. Mean occupancy by ecoregion for 1998 and 2020 can be found in *SI Appendix*, Table S4.

**Table 1.** Percent change in *B. occidentalis* occupancy from 1998 to 2020 and associated 95% credible intervals for each ecoregion (Fig. 1*A*) in the western conterminous United States

Ecoregion	% Change 1998 to 2020
Blue Mountains (BM)	−51 (−61, −38)
Cascade Range (CR)	−67 (−74, −58)
Coastal Forest (CF)	−75 (−83, −64)
Colorado Plateau and Wyoming Basin (CPWB)	−63 (−73, −51)
Columbia Plateau (CP)	−56 (−76, −25)
Great Basin (GB)	−66 (−77, −54)
Greater Yellowstone Ecosystem (GYE)	−15 (−23, −5)
Isolated Mountains in Prairie (IMP)	−36 (−46, −24)
Mediterranean California (MC)	−82 (−90, −72)
Northern Rocky Mountains (NRM)	−37 (−47, −22)
Semi-arid Prairies (SP)	−51 (−58, −43)
Sierra Nevada (SN)	−53 (−71, −26)
Southern Rocky Mountains (SRM)	−72 (−79, −62)
Uinta and Wasatch Mountains (UWM)	−40 (−50, −29)
Mogollon Rim and Mountains (MRM)	−82 (−88, −73)
Madrean Archipelago (MA)	−83 (−92, −72)

**Fig. 2.**
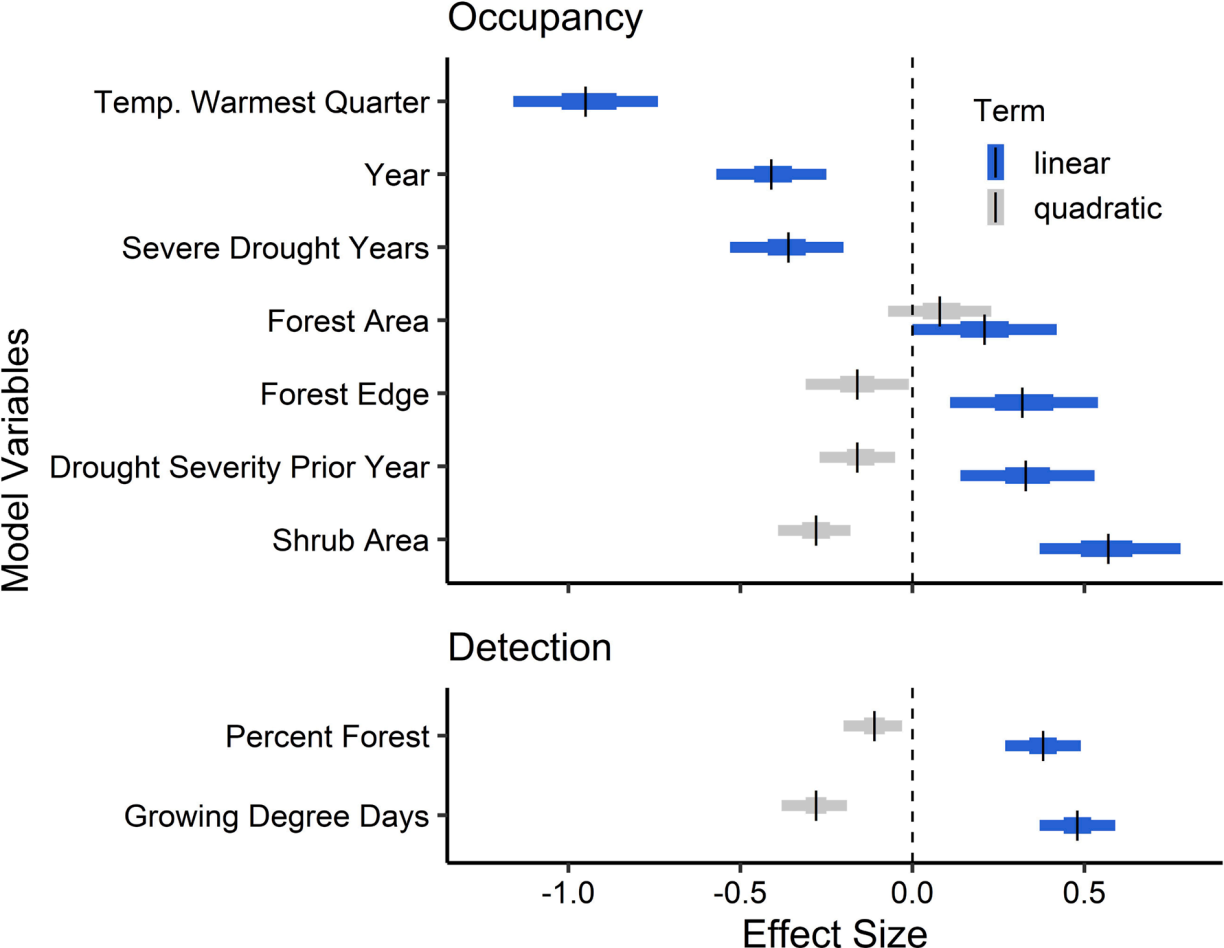
Occupancy (*Top*) and detection (*Bottom*) of *B. occidentalis-*related coefficients from the Bayesian hierarchical model. Vertical hashes indicate mean effect size. Error bars represent the 50% (thick) and 95% (thin) credible interval regions around scaled coefficients.

The final model assessing trends in *B. occidentalis* occupancy from 1998 to 2020 in the western conterminous United States included three climate and three land cover variables (Fig. 2). Of all the variables, temperature during the warmest quarter had the largest effect on occupancy with twice the negative effect as the second largest climate effect, consecutive years of severe drought (Fig. 2). Occupancy declined with increasing mean temperature during the warmest quarter, ranging from a predicted high of 0.93 at 8 °C to a low of <0.01 at 32 °C (*SI Appendix*, Fig. S1). Consecutive years of drought within the previous 5 y and increasing severity of drought (scPDSI) in the prior year also reduced occupancy for *B. occidentalis*, with some evidence that occupancy can also be lower following very wet years (negative quadratic effect in Fig. 2; see also *SI Appendix*, Fig. S1 and Table S1 for drought metric definitions). Land cover variables, serving as proxies for forage, nesting, and overwintering resources, had a complex relationship with occupancy. We found a strong nonlinear relationship between occupancy and shrub area, with higher occupancy at intermediate amounts of shrub area (~19 km^2^ per 100 km^2^, *SI Appendix*, Fig. S1). Occupancy increased with increasing forest area but had a quadratic relationship with forest edge (Fig. 2). The highest predicted occupancy occurs at 750 m of forest edge per 1 km^2^ (*SI Appendix*, Fig. S1), calculated with all other predictor variables at their mean values. A Freeman–Tukey goodness-of-fit test found no evidence of lack of fit in the final occupancy model; Bayesian *P*-value = 0.49.

**Fig. 3.**
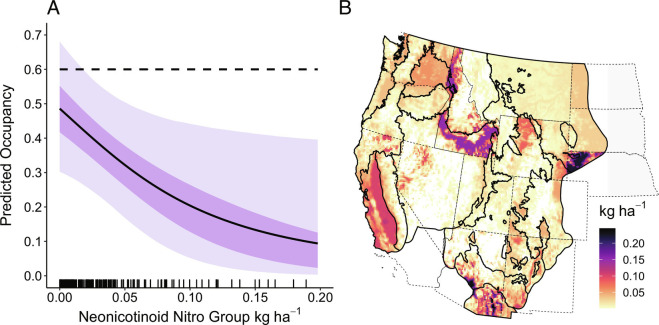
(*A*) Mean predicted occupancy for *B. occidentalis* as a function of increasing neonicotinoid (nitroguanidine group) application rate (kg ha^−1^), 2008 to 2014, in the conterminous United States (shaded ribbons indicate the 95% (light) and 50% (dark) credible interval regions). Dotted line represents mean predicted occupancy in the absence of any neonicotinoid use (0.60). Hash marks on x-axis indicate observations of varying neonicotinoid loads in observed data. (*B*) Map of mean maximum neonicotinoid application rate on croplands during 2008 to 2014 by ecoregion.

In a subset analysis of pesticide effects on *B. occidentalis* occupancy (2008 to 2014) that also included climate and land cover variables, neonicotinoid pesticides had a similar effect size as consecutive years of severe drought and ranked behind temperature during the warmest quarter for largest effect size (*SI Appendix*, Table S7). The subset analysis indicated that the mean predicted occupancy was 35% lower at sites where neonicotinoid (nitroguanidine group) applications occurred (0.39, 95% CRI = 0.24, 0.56) compared to where applications did not occur (0.60, 95% CRI = 0.39, 0.81). Within areas of neonicotinoid application, occupancy further declined as the rate of application increased (Fig. 3*A*). Ecoregions with the highest amounts of neonicotinoid application on farmland (kg ha^−1^) had the lowest occupancy by the end of the study period (Madrean Archipelago, Mogollon Rim and Mountains, Mediterranean California, Columbia Plateau, and northern portions of the Great Basin; Fig. 3*B*). Compared to results from the full 1998 to 2020 analysis, temperature during the warmest quarter had a similarly large effect size and consecutive years of drought had double the effect size in the subset model of occupancy (2008 to 2014) (*SI Appendix*, Tables S3 and S7).

Overall, the mean detection rate of *B. occidentalis* during surveys was 0.22 (95% CRI = 0.20, 0.25), indicating that when present at a site, *B. occidentalis* was only observed in an average of 2 out of 10 surveys. Cumulative growing degree days and percent forest cover strongly affected detection rates, both in a nonlinear manner (Fig. 2). These results align with those of previous research (27) and underscore the importance of accounting for imperfect detection during surveys for pollinators. Detection rates increased over the course of the season and peaked at 125 growing degree days before declining as the growing season ended (*SI Appendix*, Fig. S2). This means surveys occurring during the middle of the growing season have a better opportunity to detect *B. occidentalis* than those taking place at the beginning or end of the season. Detection also increased with increasing forest cover but declined after forest cover exceeded 52% (*SI Appendix*, Fig. S2), reflecting the increased abundance of *B. occidentalis* in preferred habitat and the influence of increasing vegetation density reducing observers’ abilities to detect individual bees during surveys.

**Fig. 4.**
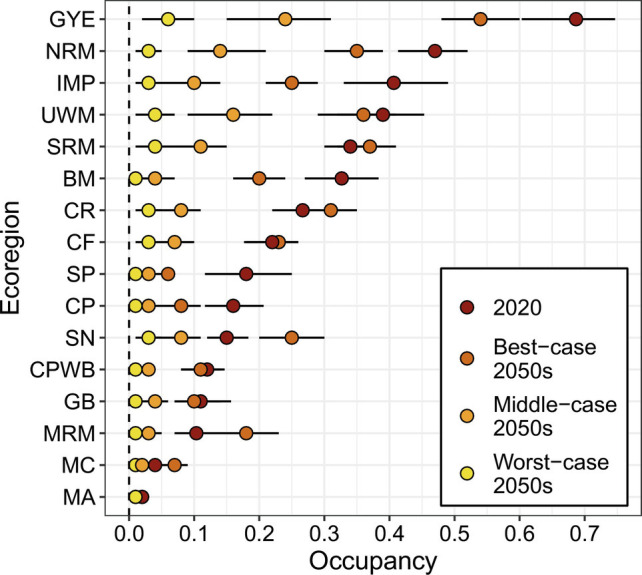
Summarized projected mean occupancy of *B. occidentalis* in 2020 and three future scenarios for the 16 ecoregions in the western conterminous United States. Error bars represent the associated 95% credible intervals. See Table 1 for abbreviation definitions and *SI Appendix* or more information on the data in this figure.

Future projections of *B. occidentalis* occupancy indicate continued declines in all modeled scenarios (Fig. 1 *D*–*F*). Under the most optimistic scenario, which includes changes to climate and land cover only and no compounding effects from other sources such as disease or pesticides, we found statistically supported declines in 44% of ecoregions (n = 7), no change in occupancy in 25% (n = 4), and increases in 31% (n = 5) (Fig. 4). However, 3 of 5 of the ecoregions with projected increases under the most optimistic scenario (Madrean Archipelago, Mogollon Rim and Mountains, and Mediterranean California) had the lowest estimated occupancy in 2020 (Fig. 4 and *SI Appendix*, Table S9), and the projected increases in those ecoregions represent very limited range-wide gains for *B. occidentalis*. Projections indicate declines in occupancy for all ecoregions under the middle- and worst-case scenarios, which do not assume that other sources of decline disappear (Fig. 4 and *SI Appendix*, Tables S10 and S11). In the middle-case scenario, the mean occupancy in 75% (n = 12) of the ecoregions is ≤0.10 and under the worst-case scenario, all ecoregions have a mean occupancy ≤0.06 (Fig. 4 and *SI Appendix*, Table S10).

## Discussion

We demonstrate the complex mechanisms leading to the precipitous decline of a once-common species and highlight the considerable challenges to the persistence of *B. occidentalis* over the next 30 y. We found large, but spatially and temporally variable declines in *B. occidentalis* associated with landscape-level changes in climate, land cover, and pesticide usage. Future occupancy projections of *B. occidentalis* into the 2050s mostly indicate continued range-wide declines in 44 to 100% of ecoregions depending on scenario. Only the most optimistic future projection indicates limited further reductions of *B. occidentalis*, and that scenario assumes that all unspecified stressors we could not explicitly incorporate in the full 1998 to 2020 model, including disease and pesticides, cease to exist or are counteracted by shifts in management actions. Given that a complete abatement of extenuating factors beyond changes in climate and land cover would be extremely challenging to achieve, the middle- and worst-case future scenarios are more plausible outcomes.

Relative to an earlier study covering 1998 to 2018 (27), we found a smaller negative trend. In addition to new survey data that, in part, occurred to fill important information gaps identified in ref. 27, several other differences strengthen the findings here. Our study includes a suite of fine-resolution explanatory variables (e.g., climate, land cover, and pesticides) that more directly represent the ecology of the species than the prior work, which had broad spatial covariates (e.g., latitude and elevation) explaining general patterns in the occurrence of *B. occidentalis* (27). Our study also treats surveys as temporal and spatial replicates of grid cells, allowing for the inclusion of 200% more survey data. Last, we defined the species range in our analysis more conservatively, thus eliminating data from the extreme periphery of the range edge where the occurrence of *B. occidentalis* is historically unlikely (e.g., portions of eastern North and South Dakota, Nebraska, and Kansas).

Recent range-wide declines in *B. occidentalis* were most strongly linked to climate conditions, particularly increasing temperature. Increased temperatures also correlate to contractions in the distribution and relative abundance of other bumble bee species globally (25, 28). Changing climate conditions can affect bumble bee populations directly by influencing bee survival and foraging activity and indirectly by altering floral resource availability (29). Higher temperatures increase the likelihood of species reaching their thermal limits, reducing their ability to forage during hot days, and *B. occidentalis* ranks among the most sensitive *Bombus* species to heat stress (30). Extended heat waves, which are expected to increase in the future (31), could be increasingly detrimental on bumble bee populations (25). We found temperature during the warmest quarter of the year to be the most informative in modeling *B. occidentalis* occupancy; however, stress due to warming temperatures can occur year-round. Increasing temperatures can affect all bumble bee life stages and queens may be especially sensitive while overwintering underground. Lab studies suggest queens experiencing warm temperatures during winter diapause drain more from their energy reserves and thus risk starvation (32). Concurrently, increasing temperatures may increase soil heat accumulation and drive earlier queen emergence, exposing queens to the increased possibility of resource shortages if their emergence occurs before flowering of key spring plants (33).

Drought also negatively affected *B. occidentalis* and can be exacerbated by increasing temperatures. Drought impacts bumble bee populations through indirect effects on floral resource availability (34), such as increases in seasonal floral resource gaps versus continuous resource availability that reduce bumble bee colony performance (35). Besides changes in floral abundance or continuity, drought can also reduce the quality and quantity of pollen and nectar resources (36). Given that food resources often limit bumble bee colony growth and reproduction (37), it is likely that such indirect drought effects on floral resources had a strong and widespread negative impact on *B. occidentalis* populations. We also found substantial variability on the effects of very wet years and posit that this arises due to potential negative effects of high-intensity precipitation. Under future climate scenarios, the frequency and intensity of short-duration (minutes to hours) extreme precipitation events may increase (38). During these events, nests may be flooded or floral resource availability and bee foraging activity could be substantially disrupted, resulting in a negative relationship between bumble bee persistence and precipitation at the high end of precipitation totals. Long-term monitoring data on bumble bee populations, climate, and floral resource conditions are essential to separate these direct and indirect effects of climate change.

Our future scenarios only project forward 30 y into the mid-century (2050s), but temperatures are expected to increase even more (1.5 to 4 °C) by the late-21st century (39), with more warming in northern than southern regions. We modeled many potential climate factors (*SI Appendix*, Table S1) that were not informative for the current trends in *B. occidentalis* but may become important as conditions exceed those measured during our study and interact in new ways, including more extreme droughts and heatwaves (31).

We found a strong, positive relationship between *B. occidentalis* occupancy and woody plant cover, specifically forest area (Fig. 2 and *SI Appendix*, Fig. S1). We also found strong nonlinear relationships for occupancy with shrub area and forest edge with highest predicted occupancy at intermediate levels for both factors (Fig. 2 and *SI Appendix*, Fig. S1). These land cover types may be overlooked in bumble bee conservation, but can often be sites of foraging, nesting, and overwintering habitat (reviewed in ref. 40). Although this species was once broadly distributed in a variety of habitats across the western United States, including grasslands, forests appear to be a significant refuge for this species, perhaps due to microsite conditions which may buffer against the effects of warming temperatures. Understanding how different management practices maintain suitable habitat within forests is essential to the conservation of this species.

Over the last 150 y, western forests have changed dramatically due to fire suppression and livestock grazing practices, as well as the ongoing effects of drought, invasive species, and climate change (41). One consequence of these landscape changes has been a vast decrease in small forest openings (<50 m in length, 42), potentially reducing the availability of understory flowering resources (documented in deciduous forests, 40). Fires can increase heterogeneity in the forest landscape, with known positive effects on bumble bee abundance (43), but the combined effects of changing fire regimes and existing forest structure on bumble bee populations are poorly understood at a landscape scale, especially outside the context of temperate deciduous forests (40). Because projections of land cover including fire did not exist at the scale of our analysis, none of our scenarios explicitly account for reductions in forest cover resulting from projected increases in fire severity, area, and length of the fire season in western forests (44, 45), suggesting that our analysis may be underestimating the role these effects have on *B. occidentalis*. Although we document relationships with total forest quantity in this study, understanding forest quality in terms of suitability for nesting, overwintering, and foraging habitat is critically important and requires targeted field studies. Focused research on nesting and overwintering ecology will be integral to conservation planning that supports persistence of this species (27). Concurrent studies to understand how adaptive management strategies (e.g., timber harvest, prescribed fire, planting floral resources, and livestock grazing) can help maintain or improve overall habitat condition will expedite identification of effective conservation measures.

Conversion of land to agriculture in North America has slowed or even reversed in recent decades, while the intensity of management on existing farmland has continued to increase, including the use of pesticides which can contribute to bee declines (46, 47). In contrast to forest cover, the quantity of agricultural land did not emerge as a significant variable influencing *B. occidentalis* occupancy. However, the increasing intensity of agricultural management, specifically the use of nitroguanidine neonicotinoid insecticides, was associated with declining *B. occidentalis* occupancy in our subset model evaluating pesticide usage. This finding aligns with the documented lethal and sublethal effects of these systemic insecticides on bees through exposure to contaminated soil, dust, guttation fluid, nectar, or pollen (48–50). Early studies of honey bees suggested that sublethal exposure to neonicotinoids can also weaken bee immunity and synergize with pathogens such as *V. bombi* (51), but evidence is lacking for this effect in bumble bees (52). Therefore, these two stressors likely act in parallel rather than synergistically, although it would be valuable to investigate their interaction in *B. occidentalis* specifically.

Previous work found positive associations of the fungicide chlorothalonil with prevalence of the pathogen *V. bombi* and negative associations of total fungicide use with the presence of four *Bombus* species, including *B. occidentalis* (53). In our pesticide analysis, neither chlorothalonil nor fungicides as a whole emerged in our most explanatory model. In prior work, McArt et al. estimated pesticide usage for a single year (2009) using county-level application rates and assessed all species in their study together as a single unit rather than as independent species (53). Our specific focus on *B. occidentalis* over a greater spatial and temporal range and use of state- and crop-specific estimates of pesticide usage may explain some of the differences between our findings and those of prior studies. We also used a different modeling approach that accounts for imperfect detection.

One additional consideration includes whether the statistical association between nitroguanidine neonicotinoids and *B. occidentalis* occupancy could be confounded with other agricultural activities. If insecticide usage was concentrated on crops that use managed pollinators, the relationship could be driven by dynamics with the parasite *V. bombi* and not insecticide use per se. However, the spatial and temporal patterns of insecticide and managed bee use have distinct signatures. While the nitroguanidine neonicotinoids have a wide array of uses in the United States, most of their use by total weight, area, and toxic load is seed treatments on field crops such as corn and soybean (54, 55) which do not use managed pollinators, but within which bees forage. The most prevalent pollinator-dependent crop across our study region was alfalfa, which contributed <2% of nitroguanidine neonicotinoid applications during the study period (56) and only requires managed bees for the small minority of acres grown for seed production (<1% of acres according to the USDA). Furthermore, the significant increase in the use of neonicotinoids began in 2004 (54), whereas the use of *B. occidentalis* as a commercial pollinator (the putative source of *V. bombi*) started in the early 1990s and ended in the early 2000s (17, 57). We, therefore, find it unlikely that the neonicotinoid effect was driven by collinearity with *V. bombi*. Instead, our findings join a substantial body of research implicating neonicotinoids (particularly the nitroguanidine group) in ongoing wild bee declines (48, 49) and demonstrate this relationship at a broad spatial scale in the United States. Current evidence suggests that use of these insecticides could be significantly curtailed without reducing crop yield and, in some cases, curtailment could even increase crop yield via insect-provided pollination services (57, 58).

### Improving Future Assessments.

Status assessments for pollinators are often hampered by inadequate spatial and temporal data (59, 60), which emphasizes the importance of using all available data sources. Data from museum records and databases can be combined with current monitoring and community science programs to inform future pollinator management and conservation priorities (5, 61). However, each data source has its own strengths and weaknesses and requires careful practice in their use and interpretation (e.g., museum records often lack information on effort or collection methods). The last decade has seen an increase in the availability of web applications, software, and community science projects geared to expand public engagement with the sciences. In addition to global initiatives to document biodiversity with community scientists (e.g., iNaturalist), regionally standardized efforts by state and nonprofit organizations are showing promise. For example, the Pacific Northwest (PNW) Bumble Bee Atlas (62) has set a standard for community science programs by training participants to collect standardized data and take photographs of bumble bees to support more accurate species identification. The in-depth training, robust quality control, and purposeful sampling design with repeat surveys of the bumble bee community allowed incorporation of these data into our occupancy analysis of *B. occidentalis.* The design and implementation of the PNW Bumble Bee Atlas provides a framework for programs that seek to inform the management of bumble bees or pollinator communities more broadly.

Even with the demonstrated importance of community science efforts, museum specimens, and data repositories in cataloging rare and declining bumble bee species, consensus on how to best survey and monitor bumble bees remains elusive. Methods to monitor bee communities, including nonnative species such as the honey bee, have been actively discussed for more than two decades (59, 60, 63, 64). Specifically, how and whether a bee-monitoring plan should be implemented at a national or international scale continues to be an active place for constructive dialog. Building consensus on consistent data collection for pollinator monitoring across government agencies and internationally could better inform scientifically defensible management and restoration activities across species. Equally important to consistent data collection is ensuring the development of a monitoring program occurs with attention toward filling spatial gaps in knowledge in an unbiased manner (27). Regardless of the state variable of interest (i.e., abundance or occupancy), accounting for imperfect detection during the analysis of these data is integral to accurately assess changes in population status and the effects of stressors like those measured in this study. Similarly, the scarcity of fine-resolution, spatially explicit information about stressors and resources at the scale of species’ ranges hinders robust assessment of the role these factors play in population trends. As examples, in the United States, we have no modern cohesive dataset on pesticides, no mapping of the progression of various diseases, and no fine-scale information on commercial beehives or livestock grazing intensities.

## Conclusions

The traditional view of generalist species is one of the robustness to spatially varying stressors; by definition, these types of species have flexible resource requirements to survive and reproduce. They exist across many ecosystems because they can persist in the face of changing environmental conditions. On evolutionary timescales, this definition of generalist species may hold true, but in the present period of rapid global change, even generalists are facing uncertain futures, as we have demonstrated. Much like the decline of the passenger pigeon, emblematic of wildlife species susceptible to the pressures of market hunting, the rapid loss of a once-common pollinator like *Bombus occidentalis* may serve as a bellwether for many other pollinators that could be similarly impacted by projected changes of temperature and precipitation in the future.

We have demonstrated how macroscale stressors, including climate change, likely drive range-wide declines in *B. occidentalis.* The extensive nature of this decline suggests that locally targeted solutions may not be sufficient to counteract declines of this and other pollinator species. Novel management strategies, such as a Resist-Accept-Direct approach (65), may be useful in determining how conservation resources should be focused to increase the likelihood of success and allow for learning from actions to inform future decision-making. Identifying where management actions could be most effective under climate change is imperative given the large changes expected to occur in the western United States during the 21st century under both the medium- (e.g., RCP 4.5) and high-end (e.g., RCP 8.5) greenhouse gas emission scenarios. For example, declines in the southern and western portions of *B. occidentalis* range may be severe and likely enough to warrant acceptance of those declines to free up resources to resist declines via management action in other geographies for maximum benefit. These types of conversations are complicated but necessary in this time of acute declines in biodiversity. As ideal climate conditions for *B. occidentalis* shift northward, international collaboration will be essential for the species’ conservation including consideration of populations in Canada, where *B. occidentalis* has been nationally assessed as threatened (66) but is still locally abundant in montane ecosystems.

## Materials and Methods

We used Bayesian occupancy modeling, accounting for imperfect detection, to estimate trends in *B. occidentalis* across its range and by ecoregion and projected occupancy under several future scenarios. For full methods, please see *SI Appendix*. We relied on data from the Bumble Bees of North America (BBNA) database which contains vouchered specimen records from a range of sources including federal and state agencies and academic, tribal, and community science efforts (67). In addition, we conducted several data calls to identify new bumble bee survey data. We used only records with complete information on survey date, location (latitude, longitude), and species observed. We retained records with multiple species recorded in a single survey or locations with at least two surveys when only a single species was recorded. We converted survey data to detection (if *B. occidentalis* was observed) or nondetection data (if *B. occidentalis* was not observed) for use in occupancy modeling.

We modeled occupancy using Bayesian hierarchical occupancy models at sites defined as 10 km × 10 km grid cells. Each site (grid cell) could have multiple survey events replicated spatially and temporally. Our dataset included 14,457 unique surveys conducted from 1998 to 2020 across 2.8 million km^2^ in the conterminous United States. We modeled occupancy as a function of 17 temperature- and precipitation-related variables (*SI Appendix*, Table S1) and eight land cover types: grassland, shrub, forest, bare ground, agriculture, litter, urban development, and wetland (*SI Appendix*). We also conducted an analysis incorporating the effects of pesticides on *B. occidentalis* occupancy using 2,288 unique surveys from 2008 to 2014. This subset analysis was temporally restricted due to limited fine-resolution crop and pesticide data (*SI Appendix*).

For each occupancy analysis, we employed a multistep forward model selection procedure within variable categories to determine the final combination of explanatory variables, adding variables sequentially based on univariate performance as measured by Watanabe–Akaike information criterion (WAIC; 68) and retaining variable combinations that reduced WAIC. We conducted all analyses using the statistical computing environment R v4.1.0 (69). We wrote occupancy models in the JAGS language (70) and fit models using package NIMBLE (71, 72). Continuous variables were standardized with a mean of 0 and a SD of 1 to allow for direct comparison of effect sizes. Priors for all effects on occupancy and detection were specified as diffuse normal distributions with a mean of 0 and a variance of 100. We ran all models using three Markov Chain Monte Carlo (MCMC) chains of 30,000 iterations each and retained 28,000 values per chain, after discarding 2,000 for adaptation and burn-in. We conducted posterior predictive checks and evaluated the fit of the final models from which we made our inference using a Freeman–Tukey discrepancy goodness-of-fit (GOF) test, generating a Bayesian *P*-value (73).

We created annual predicted spatial surfaces of occupancy and summarized trend as percent change in occupancy across 16 ecoregions from 1998 to 2020 (Fig. 1*A*). We considered statistical support for trends sufficient when 95% credible intervals did not overlap zero. Ecoregions were delineated a priori by the U.S. FWS as part of the prelisting process for the Species Status Assessment of *B. occidentalis* using biophysical features and expert input. We also projected future *B. occidentalis* occupancy to the mid-century (2050 to 2059), including changes in climate, land cover, and overall trend momentum across a range of plausible scenarios (*SI Appendix*, Table S8). For each future scenario, we generated annual projected occupancy surfaces for 2050 to 2059 and averaged these across the species’ extent and within ecoregions to compare to the predicted occupancy in 2020 to estimate percent change into the future.

## Supplementary Material

Appendix 01 (PDF)

## Data Availability

Bumble bee occupancy data are available online via the BBNA dataset (67, https://doi.org/10.5061/dryad.c59zw3r8f). Additional bumble bee data collected in Montana, North Dakota, South Dakota, and Nevada are available in a USGS data release (74, https://doi.org/10.5066/P931YWY8). Also available via USGS data releases are spatial layers for western bumble bee occupancy in the western conterminous United States for 1998, 2020, and the three 2050’s future scenarios (75, https://doi.org/10.5066/P9UHMCV1) as well as estimated annual nitroguanidine neonicotinoid application rates for the western United States from 2008 to 2014 (76, https://doi.org/10.5066/P9H45NUG).
