# Supplementary material for: Recent and future declines of a historically widespread pollinator linked to climate, land cover, and pesticides
Source: Proc Natl Acad Sci U S A. 2023 Jan 23;120(5):e2211223120. doi: 10.1073/pnas.2211223120 (PMC9945941; doi:10.1073/pnas.2211223120)
Supplement: Supplementary file 1 — Appendix 01 (PDF) [file pnas.2211223120.sapp.pdf]

PNAS

Supplementary Information for
Recent and future declines of a historically widespread pollinator linked to climate, land cover, and pesticides

William M. Janousek[1†*], Margaret R. Douglas[2], Syd Cannings[3], Marion A. Clément[4], Casey M. Delphia[5], Jeffrey G. Everett[6], Richard G. Hatfield[7], Douglas A. Keinath[4], Jonathan B. Uhuad Koch[8], Lindsie M. McCabe[8], John M. Mola[9], Jane E. Ogilvie[10], Imtiaz Rangwala[11], Leif L. Richardson[7], Ashley T. Rohde[8], James P. Strange[12], Lusha M. Tronstad[13], Tabitha A. Graves[1†]

1. U.S. Geological Survey, Northern Rocky Mountain Science Center, West Glacier, MT, USA.
2. Department of Environmental Studies & Environmental Science, Dickinson College, Carlisle, PA, USA.
3. Canadian Wildlife Service, Environment and Climate Change Canada, Whitehorse, YT, Canada.
4. U.S. Fish and Wildlife Service, Cheyenne, WY, USA.
5. Montana State University, Bozeman, MT, USA.
6. U.S. Fish and Wildlife Service, Portland, OR, USA.
7. Xerces Society for Invertebrate Conservation, Portland, OR, USA.
8. U.S. Department of Agriculture - Agricultural Research Service - Pollinating Insect Biology, Management, Systematics Research Unit, Logan, UT, USA.
9. U.S. Geological Survey, Fort Collins Science Center, Fort Collins, CO, USA.
10. Rocky Mountain Biological Laboratory, Crested Butte, CO, USA.
11. North Central Climate Adaptation Science Center & Cooperative Institute for Research in Environmental Sciences, University of Colorado Boulder, Boulder, CO, USA.
12. Department of Entomology, The Ohio State University, Columbus, OH, USA.
13. Wyoming Natural Diversity Database, University of Wyoming, Laramie, Wyoming, USA.

[†]Denotes Co-lead Authors

Corresponding Author: *William M. Janousek
Email: wjanousek@usgs.gov

This PDF file includes:

Supplementary text
Figures S1 to S2
Tables S1 to S11
SI References

Supplementary Information Text

The supplemental information provided here covers additional details on the occupancy analyses for *Bombus occidentalis*, the modeling approaches used, and additional results, figures, and tables.

Occupancy Analysis 1998 - 2020

Bumble bee, climate, and land cover data We used data from the Bumble Bees of North America (BBNA) database (1). We also incorporated additional bumble bee survey data collected in Montana and the Dakotas (2). We inferred non-detection of *B. occidentalis* from available survey data with the following assumptions: the reporting of bumble bee detections was consistent across space and time, the presence of *B. occidentalis* was recorded if observed, and variation in sampling method and effort across studies had minimal impact on the detection process overall.

We used a 10 km x 10 km spatial grid applied across the range of *B. occidentalis* in the conterminous United States to delineate unique sites used in our occupancy analysis. Each grid cell from the 10 km resolution grid is treated as a site. The size of sites was chosen to match to the estimated maximum dispersal of *B. occidentalis* based on related *Bombus* spp. (3,4). We treated individual bumble surveys as temporal and spatial replicate surveys of sites.

We derived precipitation- and temperature-related explanatory variables from DAYMET v.4 (5) and four drought-related explanatory variables from PRISM (6). Definitions of climate variables are provided in Table S1. We used land cover data from the Rangeland Analysis Platform (RAP, 7). RAP consists of percent cover estimates for grassland, shrub, forest, bare ground, agriculture, litter, and urban development categories. We converted RAP land cover categories from percent cover to area for each 10 km x 10 km grid cell. We used a binarized layer of RAP forest cover, upscaled to 1 km resolution, to calculate the amount of forest edge within each site using the *landscapemetrics* package in program R v4.1.0 (8,9). We also obtained wetland area from the USFWS National Wetlands inventory (10). We summarized all spatial variables at the site-level (i.e., across each grid cell).

Occupancy Model We used Bayesian hierarchical occupancy models to assess site-level occupancy of *B. occidentalis* (11). There was insufficient site-level replication from one year to the next across the full 23-year period of the study to allow for a dynamic occupancy model. We modeled the true state of occupancy (z) at site *i* in year *j* as a Bernoulli random variable with probability Ψ_{ij}:

$$z_{ij} \sim Bern(\Psi_{ij})$$

where z_{ij} = 1 if the species is present during sampling at site *i* in year *j*, and zero otherwise. Observations from sampling occasions, y_{ijk}, denote detection or non-detection of species at site *i* in year *j* during sampling occasion *k*. We modeled site-level species occupancy probabilities, Ψ_{ij}, as a function of an intercept ($\alpha 0$) and *X* site-specific explanatory covariates:

$$logit(\Psi_{ij}) = \alpha 0 + \alpha 1_{ij} + \cdots + \alpha X_{ij}.$$

We used a continuous linear year effect to evaluate changes in *B. occidentalis* occupancy from 1998 to 2020, and this variable was present during selection of all other explanatory variables. Species detection was modeled as a Bernoulli random variable:

$$y_{ijk} \sim Bern(p_{ijk,}, z_{ij})$$

where p_{ijk} represents the detection probability of a species at site *i* in year *j* during sampling occasion *k*. We modeled survey-level species detection probabilities, p_{ijk}, as a function of an intercept ($b0$) and *X* survey-specific explanatory covariates:

$$logit(p_{ijk}) = b0 + b1_{ijk} + \cdots + bX_{ijk}.$$

JAGS Occupancy Model Example Code

```
nimbleCode({
# Occurrence Intercept Priors
        a0.mean ~ dunif(0,1)
        mu.a0 <- log(a0.mean) - log(1 - a0.mean)

# Detection Intercept Priors
        b0.mean ~ dunif(0,1)
        mu.b0 <- log(b0.mean) - log(1 - b0.mean)

# Explanatory Covariate Priors
        for(i in 1:n.covs.occ){ a[i] ~ dnorm(0, 0.01)} # Occupancy
        for(i in 1:n.covs.det){ b[i] ~ dnorm(0, 0.01)} # Detection

# Likelihood
# Ecological Process
for (i in 1:n.sites) {logit(psi[i]) <- mu.a0 + a[1]*ShrubA[i] + a[2]*pow(ShrubA[i], 2) +
                                a[3]*ForestA[i] + a[4]*pow(ForestA[i], 2) +
                                a[5]*Edge[i] + a[6]*pow(Edge[i], 2) +
                                a[7]*TwarmQ[i] + a[8]*SevDrght5Year[i]  +
                                a[9]*LagscPDSI[i] + a[10]*pow(LagscPDSI[i], 2) + a[11]*Year[i]
                z[i] ~ dbern(psi[i])}

# Observation Process – using nested indexing
for (k in 1:n.obs) {logit(p[k]) <- mu.b0 + b[1]*GDD[k] + b[2]GDD2[k] +
                                b[3]*Forest[k] + b[4]*Forest2[k]
                mu.p[k] <- p[k] * z[site[k]]
                y[k] ~ dbern(mu.p[k])}       })
```

The abundance within bumble bee colonies changes seasonally, and this can affect detection rates during surveys that occur over the course of a sampling year (12). Therefore, we included a measure of cumulative growing degree days as linear and quadratic terms in the detection portion of the hierarchical model. A growing degree day is defined as having a max temperature ≥ 55°F (12.8 °C). No specific temperature minimum threshold has been identified for *B. occidentalis* however research suggests pollinator activity declines below 50-60°F (10-15.6°C), depending on species (13,14). We used the midpoint (55°F, 12.8°C) as the assumed cutoff for foraging activity. Detection of bumble bees during surveys can also be influenced by the density of vegetation. For example, surveys in closed canopy forests tend to have lower detection rates than surveys in other land cover types (12). Consequently, we also included percent forest canopy cover as an explanatory variable in the observation process. All detection covariates were measured at the survey level.

We conducted model selection via a four-step procedure. We grouped explanatory variables into categories that represent distinct ecological and/or physical processes (temperature-based metrics, drought- and precipitation-based metrics, and land cover). We implemented variable reduction techniques by fitting univariate models for all variables, including linear and quadratic forms, and excluded variables with poor fit (i.e., 95% CRIs overlapping zero) or high collinearity with other variables (r ≥ 0.60). There is no consensus about model selection

methodology for Bayesian hierarchical models (15). We used the Watanabe-Akaike information criterion (WAIC), which is recommended when the goal is prediction (16,17). We selected between linear only or quadratic forms of individual variables from the univariate models by choosing those with lower WAIC values. We then used forward step selection, beginning with the single univariate predictor with the lowest WAIC, adding variables sequentially and retaining variable combinations that further reduced WAIC (Table S2). The variables retained in the top model from each of the three categories (temperature, precipitation, and land cover) were combined into a single final model from which we make our inference (Table S3). All variables were mean zero standardized to allow for direct comparison of effect size in the final model combination.

Some variables were excluded following the univariate assessments as part of the variable reduction process and were not included in the forward step selection process. Abnormal Frost Days fit poorly with 95% CRIs overlapping zero in the univariate model stage and was excluded from subsequent steps. In situations of high collinearity among explanatory variables we retained those with the largest effect size. The variables Diapause Warming Events and Temperature Coldest Quarter were excluded due to high collinearity with all other temperature related variables (r = 0.63-0.74 and 0.60-0.76, respectively). We found high correlations (r = 0.76-0.99) among all precipitation variables, and only included Spring Precipitation at the variable selection step. Grassland area was the only land cover category excluded from variable selection due to high collinearity with forest area (r = -0.65).

Spatial layers of western bumble bee predicted occupancy across the western conterminous United States for 1998 and 2020 are available via the USGS data release (18).

Occupancy Sub-analysis 2008-2014: Effects of Pesticides

We conducted a subset analysis to investigate the effects of pesticides on *B. occidentalis* occupancy in the conterminous U.S. between 2008-2014. This analysis was conducted under a constrained time period because pesticide data were only recorded consistently up to 2014 (19) and prior to 2008 fine resolution crop information was not available for the entire study region (20). We evaluated three broad pesticide types (Fungicides, Herbicides, and Insecticides) as well as more specific subtypes: the fungicide chlorothalonil and neonicotinoid insecticides (nitroguanidine and cyanoimine groups). Although we quantified most pesticides as application rate (kg/ha), we also tested aggregate measures of insecticide use translated into toxic loading via lethal doses (i.e., honey bee LD50s/ha). Pesticide estimates for combinations of crop, state, and year (21) were applied to the landscape by reclassifying land cover data from CropScape at 30 m resolution (20). In total, this sub-analysis considered the effects of 12 different measures of pesticides. Pesticide application intensity was summarized using the maximum observed rates within individual 10 km x 10 km sites. We used the maximum application rate in this analysis because it had better statistical properties in our dataset and because we were unable to control for 1) the rate of persistence of pesticides in the environment, 2) the potential for pesticides to travel outside of areas of known use (i.e., spillover from adjacent crop fields), or 3) the application of pesticides outside of croplands within each grid cell. For neonicotinoids the maximum application rates are correlated to the mean application rates on cropland with correlations of 0.70, 0.62, 0.54, 0.68, 0.64, 0.74, and 0.73, from 2008-2014, respectively.

We used the same model structure as the final 1998-2020 occupancy analysis with the addition of a two-level intercept denoting the presence or absence of pesticides at a site. We also used the same approach to model selection. We conducted univariate assessments and carried

forward variables with the lowest WAIC from each pesticide type (Table S5) and then combined variables sequentially beginning with the variable with the lowest WAIC to form a final model combination (Table S6). A single pesticide measure, application rate of neonicotinoids (nitroguanidine group), was retained after the variable selection process (Table S6). We then combined all variables from the final 1998-2020 model with the neonicotinoid variable to directly compare the effects of pesticides, climate, and land cover (Table S7). Mean occupancy was lower in the presence of neonicotinoids (intercept = -0.42, 95% CRI = -1.12, 0.27) and the effect of increasing neonicotinoid (nitroguanidine group) application on *B. occidentalis* occupancy from 2008-2014 was negative (-0.64, 95% CRI = -1.37, -0.02). We discuss these results in more detail in the main text. Spatial layers for annual estimated neonicotinoid application rate across the western United States from 2008 to 2014 are available via USGS data release (22).

Occupancy Future Projections to 2050s

Climate and land cover data For future projections of *B. occidentalis* occupancy (2050-2059), we used the MACAv2-METDATA dataset, which is a collection of 20 global climate models covering the conterminous USA (23). MACAv2-METDATA is downscaled based on training data (1979-2012) to reduce bias and improve spatial matching of climate projections (23). We incorporated land cover change using the U.S. Geological Survey Conterminous Land Cover Projections dataset (USGS-CLC). The USGS-CLC data are categorical land cover data at 250 m resolution and provides several land cover change projections based on range of emissions scenarios and forecasted resource consumption practices (24,25).

Occupancy Projections We created occupancy surfaces based on projected data layers for multiple climate, land cover, and trend momentum scenarios. We generated three likely future scenarios for *B. occidentalis* ranging from best- to worst-case based on the relationships of explanatory covariates to occupancy determined from the contemporary occupancy model evaluating trends in occupancy from 1998 to 2020. We used the MACA climate toolbox future climate scatter tool (26) to visualize the changes in precipitation and temperature across the western conterminous U.S. We selected climate and emission scenarios that 1) had low standard error across the range of *B. occidentalis* (27) and 2) ranged from drier and much warmer to wetter with less warming (Table S8). We assessed the effects of three different land cover projections (USGS-CLC data) on future occupancy absent changes in climate to rank the land cover projections in terms of favorability to *B. occidentalis* in the future. We then paired the land cover projections with climate scenarios from best (most favorable) to worst (least favorable) (Table S8).

Our initial model on occupancy 1998 to 2020 included a term (year) to account for sources of trend in occupancy that we could not model explicitly or account for via climate- and land cover-related effects, referred to as un-specified sources of variation (e.g., pathogens, pesticides, fine-scale floral resources). We incorporated levels of continued change into the future scenarios by modulating the strength of the year effect (Table S8). The most optimistic scenario assumed these un-specified sources of variation did not continue in the future; the year effect was held constant as if it was 2020. Changes in occupancy in the most optimistic scenario were thereby a reflection of mid-century changes in climate and land cover alone. Under the middle scenario, the year was allowed to update regularly, and the slope of the year effect was halved allowing for a reduction in but continued influence of the un-specified sources of variation in the future. The worst-case scenario assumed that these un-specified sources would continue to

influence occupancy unabated in the future, the year was allowed to update regularly, and slope of the year effect remained unchanged.

Projected land cover data (USGS-CLC) used in the future projection of *B. occidentalis* occupancy differ in resolution and specificity from the land cover data (RAP) used in the contemporary model for *B. occidentalis* occupancy in 1998-2020. The USGS-CLC data are coarser and provide land cover information on a categorical basis per pixel whereas the RAP data provide a percent cover per pixel for each land cover type. Both sources of data can be converted to land cover area per site and though correlated (r = 0.40-0.85) for the study area, their differences make them challenging to compare directly. For this reason, we refit the contemporary occupancy model for *B. occidentalis* (1998-2020) using the UGSG-CLC land cover data instead of RAP and made future projections based on this refit model (2050-2059). Refitting the model allowed us to project future trends in occupancy using a consistent source of land cover data, thus removing effects induced by differences in summarizing percent versus categorical representations of land cover. Predicted occupancy estimates in 2020 from the refit model changed very little from the original model fit (Table S9). We generated annual predicted surfaces of *B. occidentalis* occupancy for 2050 to 2059 and averaged across these annual layers to create a single mean projected occupancy for the 2050s. We averaged across the 10 annual layers (2050-2059) to account for inter-annual variability in the projected climate data. We calculated the mean occupancy within each ecoregion (Table S10) and percent change in occupancy from 2020 to the 2050s (Table S11) following the methods used in the contemporary trend estimates. Spatial layers of projected occupancy of western bumble bee across the western conterminous United States for the three 2050s future scenarios are available via the USGS data release (18).

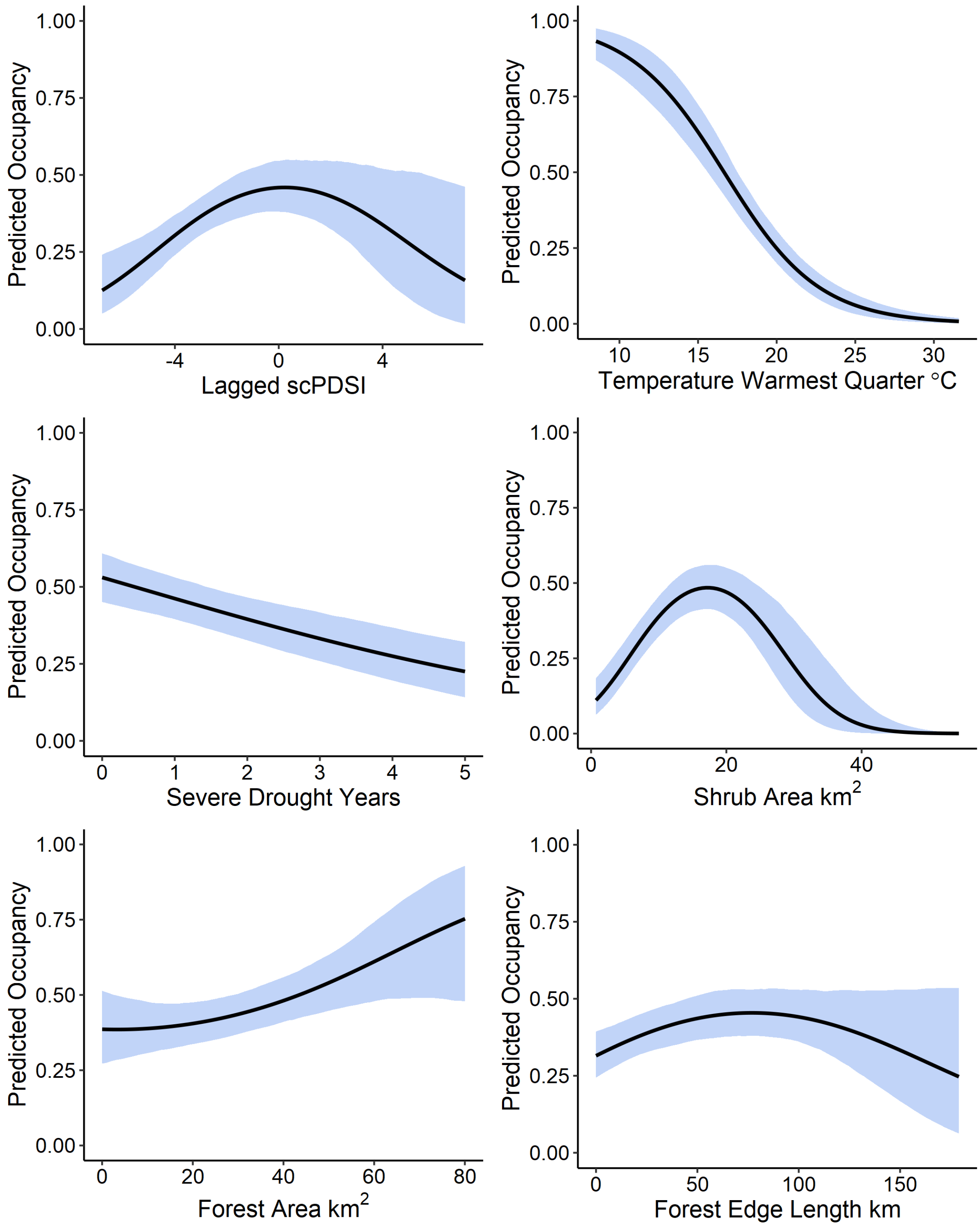

Figure S1. Mean predicted occupancy and 95% credible interval regions as a function of the six explanatory variables from the *Bombus occidentalis* occupancy analysis, 1998-2020, in the western conterminous United States.

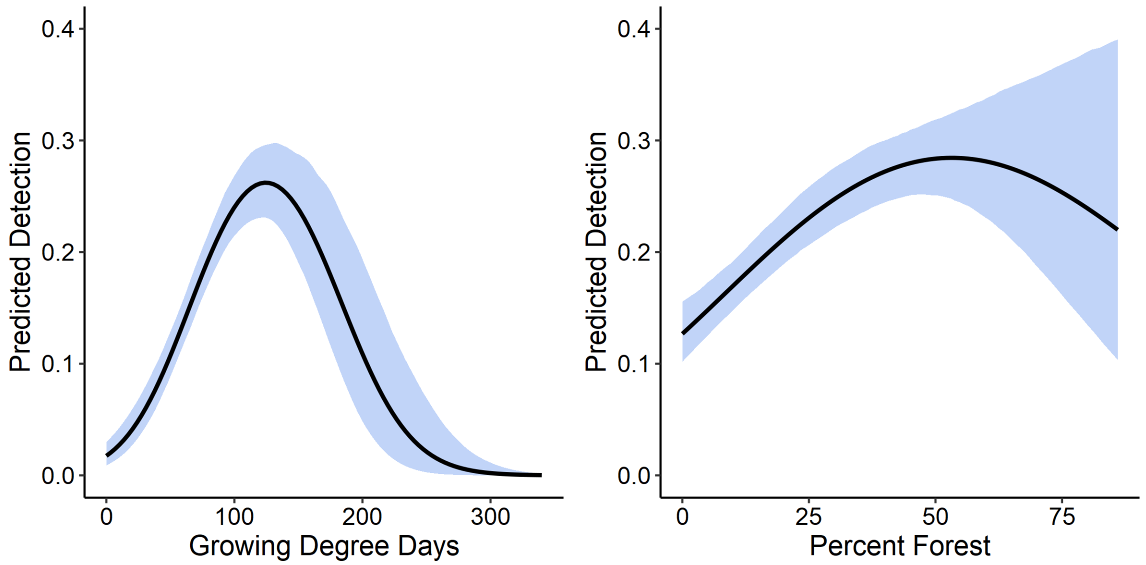

Figure S2. Mean predicted detection probabilities and 95% credible interval regions as a function of detection related explanatory variables from the occupancy analysis of *Bombus occidentalis*, 1998-2020, in the western conterminous United States.

Table S1. List of climate-related hypothesized explanatory variables considered in models *of Bombus occidentalis* occupancy 1998-2020 in the western conterminous United States and corresponding definitions.

Variable	Definition
**Temperature**	
Diurnal Range (Bioclim #2)	Mean (daily maximum – minimum temperatures)
Isothermality (Bioclim #3)	Mean (monthly (max temp. – min temp.) / (max temp. of warmest month – min temp. of coldest month))
Diapause Warming Events	Days with temperature > 55F (October to March prior to sampling)
Min Temp. vs Historical	Mean (monthly min temp. of sampling year – mean historical monthly min temp. (1980-1997))
Coldest Quarter Mean	Mean (daily temp. of coldest quarter)
Abnormal Spring Frost	Days below 32F after start of spring (SOS defined when weekly average temp. ≥ 55F)
Driest Quarter Mean	Mean (daily temp. of driest quarter)
Warmest Quarter Mean	Mean (daily temp. of warmest quarter)
Heatwave Days	Days over average historical max temp. during warmest quarter
Max Temp. vs Historical	Mean (monthly max temp. of sampling year – mean historical monthly max temp. (1980-1997))
**Drought**	
1-year Lagged scPDSI (Drought Severity)	Raw minimum self-calibrating Palmer Drought Severity Index (scPDSI) from year prior to sampling
Severe Drought Years (short-term)	Consecutive years of severe drought (scPDSI value < -3, prior 0-5 years)
Severe Drought Years (long-term)	Consecutive years of severe drought beginning 1959
Severe Drought Recovery	Years since last severe drought
**Precipitation**	
Winter Total	Total precipitation November – February prior to sampling
Winter + Spring Total	Total precipitation November – May prior to sampling
Spring Total	Total precipitation March – May prior to sampling

Temp. = Temperature

Table S2. Forward step selection tables for variable selection in *Bombus occidentalis* occupancy models, 1998-2020 in the western conterminous United States. Highlighted variable configurations from each variable set were carried forward to final analysis. Asterisk next to variables indicates both linear and quadratic forms of variables were included in configuration.

Forward Step Selection Tables

Precipitation/Drought Models	WAIC
Severe Drought Years (5 max)	4331.86
Sev. Drought Years (5 max) + Total Consecutive Sev. Drought Years	4332.87
**Sev. Drought Years (5 max) + Lag scPDSI***	**4331.59**
Sev. Drought Years (5 max) + Lag scPDSI* + Spring Precip.*	4337.59
Sev. Drought Years (5 max) + Lag scPDSI* + Years Since Sev. Drought Recovery	4361.02

Temperature Models	WAIC
**Temp Warm Q.**	**4303.63**
Temp Warm Q. + Isothermality	4327.71
Temp Warm Q. + Max Temp vs Historical	4311.58
Temp Warm Q. + Diurnal Temp Range	4320.08
Temp Warm Q. + Heatwave Days	4310.69
Temp Warm Q. + Min Temp vs Historical	4320.21
Temp Warm Q. + Temp Dry Q.*	4312.43

Land Cover Models	WAIC
Shrub Area*	4297.87
Shrub Area* + Forest Area*	4271.69
Shrub Area* + Forest Area* + Crop/Hay Area*	4271.97
Shrub Area* + Forest Area* + Bare Area*	4278.09
**Shrub Area* + Forest Area* + Total Forest Edge***	**4265.71**
Shrub Area* + Forest Area* + Total Forest Edge* + Wetland Area	4269.47
Shrub Area* + Forest Area* + Total Forest Edge* + Litter Area*	4278.62
Shrub Area* + Forest Area* + Total Forest Edge* + Developed Area	4292.61

Table S3. Estimated coefficients and 95% credible intervals of occupancy for *Bombus occidentalis*, 1998-2020 in the western conterminous United States for the most supported model. This information was used to generate Figure 2 in the main text.

Parameter	Linear Effect	Quadratic Effect
**Occupancy**		
Intercept	-0.35 (-0.64, -0.06)	
Forest Area	0.21, (0.00, 0.42)	0.08, (-0.07, 0.23)
Forest Edge	0.32, (0.11, 0.54)	-0.16, (-0.31, -0.01)
1-year Lagged scPDSI	0.33, (0.14, 0.53)	-0.16, (-0.27, -0.05)
Severe Drought Years (prior 5 years)	-0.36, (-0.53, -0.20)	
Shrub Area	0.57, (0.37, 0.78)	-0.28, (-0.39, -0.18)
Temp. Warm Quarter	-0.95, (-1.16, -0.74)	
Year	-0.41, (-0.57, -0.25)	
**Detection**		
Intercept	-1.24 (-1.38, -1.09)	
Growing Degree Days	0.48, (0.37, 0.60)	-0.28, (-0.37, -0.19)
Percent Forest	0.38, (0.27, 0.49)	-0.11, (-0.2, -0.03)

Table S4. Predicted occupancy, percent change in occupancy, and credible intervals for each ecoregion (Figure 1A) for *B. occidentalis*, 1998-2020, in the western conterminous United States. Portions of this table appear in the main text (Table 1).

Ecoregion	1998 Occupancy	2020 Occupancy	% Change
Blue Mountains	0.68 (0.57, 0.79)	0.33 (0.28, 0.38)	-51 (-61, -38)
Cascade Range	0.63 (0.52, 0.75)	0.21 (0.18, 0.23)	-67 (-74, -58)
Coastal Forest	0.55 (0.41, 0.71)	0.13 (0.11, 0.17)	-75 (-83, -64)
Colorado Plateau and Wyoming Basin	0.43 (0.34, 0.53)	0.16 (0.12, 0.20)	-63 (-73, -51)
Columbia Plateau	0.27 (0.16, 0.40)	0.11 (0.08, 0.15)	-56 (-76, -25)
Great Basin	0.47 (0.35, 0.58)	0.16 (0.11, 0.22)	-66 (-77, -54)
Greater Yellowstone Ecosystem	0.86 (0.76, 0.93)	0.73 (0.66, 0.80)	-15 (-23, -5)
Isolated Mtns in Prairie	0.64 (0.52, 0.75)	0.41 (0.34, 0.48)	-36 (-46, -24)
Mediterranean California	0.24 (0.17, 0.33)	0.04 (0.03, 0.06)	-82 (-90, -72)
Northern Rocky Mountains	0.77 (0.64, 0.87)	0.48 (0.43, 0.54)	-37 (-47, -22)
Southern Rocky Mountains	0.73 (0.65, 0.81)	0.36 (0.32, 0.40)	-51 (-58, -43)
Semi-arid Prairies	0.29 (0.20, 0.42)	0.14 (0.09, 0.20)	-53 (-71, -26)
Sierra Nevada	0.49 (0.42, 0.57)	0.14 (0.11, 0.17)	-72 (-79, -62)
Uinta and Wasatch Mtns	0.73 (0.62, 0.83)	0.43 (0.38, 0.50)	-40 (-50, -29)
Mogollon Rim and Mountains	0.34 (0.27, 0.41)	0.06 (0.04, 0.09)	-82 (-88, -73)
Madrean Archipelago	0.10 (0.06, 0.16)	0.02 (0.01, 0.03)	-83 (-92, -72)

Table S5. Results from univariate assessments for each pesticide type considered in the sub-analysis of the effects of pesticides on *Bombus occidentalis* occupancy, 2008-2014, in the western conterminous United States. Highlight indicates top performing variables from each pesticide type which were then compared in a forward step selection process (Table S6). Summary metrics that included the presence of neonicotinoids were categorized under the Insecticide group. All variables measured as maximum application rate per 10 km x 10 km grid cell.

	**Univariate Pesticide Analysis**	
**Pesticide Type**	**Coefficient**	**WAIC**
Fungicide		
**All Fungicides**	**-1.01, (-1.49, -0.61)**	**898.78**
Chlorothalonil	-0.95, (-1.35, -0.56)	899.18
Herbicide		
**All Herbicides**	**-0.56, (-1.05, -0.19)**	**897.78**
Insecticide		
**Neonic Nitro Group**	**-1.24, (-1.79, -0.72)**	**892.47**
Neonic All Groups	-1.14, (-1.68, -0.67)	892.81
Total Toxic Load (Contact + Oral)*	-0.89, (-1.36, -0.48)	893.49
Toxic Load (Contact)	-0.64, (-1.00, -0.32)	895.05
Toxic Load (Oral)	-1.02, (-1.47, -0.6)	895.25
All Pesticides (Fungicide + Herbicide + Neonic)	-0.87, (-1.25, -0.51)	896.46
Neonic Cyano Group	-0.57, (-1.05, -0.17)	899.72
Binary Presence (Fungicide + Neonic)	-0.97, (-1.66, -0.34)	903.53
Binary Presence (Toxic Load Contact + Oral)	-1.01, (-1.84, -0.27)	906.75

*Indicates summary variable using sum of all insecticides translated into honey bee toxic doses
Neonic = Neonicotinoid, Nitro = Nitroguanidine, Cyano = Cyanoimine

Table S6. Forward selection of top variables (Table S5) from each pesticide type considered in the sub-analysis of the effects of pesticides on *Bombus occidentalis* occupancy, 2008-2014, in the western conterminous United States. Starting with the Neonicotinoid Nitroguanidine Group. No additional pesticide types improved model fit. All variables measured as maximum application rate per 10 km x 10 km grid cell.

Forward Selection Pesticides	
**Model Combination**	**WAIC**
**Neonic Nitro Group**	**892.47**
Neonic Nitro Group + All Herbicides	893.72
Neonic Nitro Group + All Fungicides	896.59

Neonic = Neonicotinoid, Nitro = Nitroguanidine

Table S7. Estimated coefficients and 95% credible intervals from the 2008-2014 subset model of occupancy for *Bombus occidentalis* in the western conterminous United States, including the effects of Neonicotinoids Nitroguanidine Group in combination with the variables from the most supported model from the larger 1998-2020 analysis (Table S3).

Parameter	Linear Effect	Quadratic Effect
**Occupancy**		
Intercept – Neonicotinoids Absent	0.46, (-0.42, 1.47)	
Intercept – Neonicotinoids Present	-0.42, (-1.12, 0.27)	
Neonicotinoids Nitroguanidine Group	-0.64, (-1.37, -0.02)	
Temp. Warm Quarter	-0.97, (-1.57, -0.44)	
Severe Drought Years (prior 5 years)	-0.63, (-1.22, -0.12)	
1-year Lagged scPDSI	-0.24, (-0.91, 0.35)	0.33, (0.14, 0.53)
Shrub Area	0.18, (-0.37, 0.75)	-0.10, (-0.39, 0.20)
Forest Area	0.17, (-0.40, 0.69)	0.26, (-0.17, 0.91)
Forest Edge	0.43, (-0.13, 1.02)	-0.61, (-1.05, -0.21)
Year	-0.01, (-0.37, 0.34)	
**Detection**		
Intercept	-0.59, (-0.89, -0.28)	
Growing Degree Days	0.74, (0.45,1.04)	-0.64, (-0.95, -0.37)
Percent Forest	0.12, (-0.15, 0.40)	-0.33, (-0.59, -0.08)

Table S8. Scenarios used in projecting *Bombus occidentalis* occupancy to mid-century (2050-2059) based upon combinations of projected climate (26) and land cover change (25). Trend momentum describes the approach to incorporating the effect of year from the contemporary occupancy model into scenarios. The year effect accounts for changes in trend that are above and beyond the changes explained directly by climate and land cover effects. Future climate models project increasing temperature in all scenarios and increasing precipitation in most scenarios.

	**Most Optimistic**	**Middle-case**	**Worst-case**
Model	CNRM-C5	CSIRO-Mk3-6-0	HAD-GEM2-ES36
RCP	4.5	4.5	8.5
Temperature change	+4.03 F	+6.64 F	+8.55 F
Precipitation change	+1.60 in.	+0.91 in.	-0.18 in.
USGS-CLC Land cover model	B1	B2	A1B
Trend momentum (Year effect)	None – all changes due solely to changes in climate and land cover. Year effect held at 2020.	Rate of decline half that predicted from 1998-2020 model.	Rate of decline predicted from 1998-2020 continues unabated.

Table S9. Comparison of the original predicted occupancy in 2020 for *Bombus occidentalis* to predicted occupancy based on each of the refit USGS-CLC land cover models.

	2020 Original Fit	Refit 2020 Occupancy USGS Land Cover		
**Ecoregion**	**RAP Land Cover**	**B1**	**B2**	**A1B**
Blue Mountains	0.33 (0.28, 0.38)	0.33 (0.27, 0.38)	0.32 (0.27, 0.38)	0.33 (0.27, 0.39)
Cascade Range	0.21 (0.18, 0.23)	0.27 (0.22, 0.29)	0.26 (0.22, 0.29)	0.27 (0.22, 0.30)
Coastal Forest	0.13 (0.11, 0.17)	0.22 (0.17, 0.26)	0.22 (0.18, 0.26)	0.22 (0.18, 0.26)
Colorado Plateau and Wyoming Basin	0.16 (0.12, 0.20)	0.12 (0.08, 0.14)	0.12 (0.08, 0.15)	0.12 (0.08, 0.15)
Columbia Plateau	0.11 (0.08, 0.15)	0.16 (0.12, 0.21)	0.16 (0.12, 0.20)	0.16 (0.11, 0.21)
Great Basin	0.16 (0.11, 0.22)	0.11 (0.07, 0.15)	0.11 (0.07, 0.16)	0.11 (0.07, 0.16)
Greater Yellowstone Ecosystem	0.73 (0.66, 0.80)	0.69 (0.61, 0.75)	0.68 (0.60, 0.74)	0.69 (0.60, 0.75)
Isolated Mtns in Prairie	0.41 (0.34, 0.48)	0.40 (0.33, 0.48)	0.41 (0.33, 0.49)	0.41 (0.33, 0.50)
Mediterranean California	0.04 (0.03, 0.06)	0.04 (0.02, 0.05)	0.04 (0.02, 0.05)	0.04 (0.02, 0.05)
Northern Rocky Mountains	0.48 (0.43, 0.54)	0.47 (0.42, 0.52)	0.47 (0.41, 0.52)	0.47 (0.41, 0.52)
Southern Rocky Mountains	0.36 (0.32, 0.40)	0.34 (0.30, 0.37)	0.34 (0.30, 0.37)	0.34 (0.30, 0.38)
Semi-arid Prairies	0.14 (0.09, 0.20)	0.18 (0.12, 0.24)	0.18 (0.12, 0.25)	0.18 (0.11, 0.26)
Sierra Nevada	0.14 (0.11, 0.17)	0.15 (0.12, 0.18)	0.15 (0.12, 0.18)	0.15 (0.12, 0.19)
Uinta and Wasatch Mtns	0.43 (0.38, 0.50)	0.39 (0.32, 0.45)	0.39 (0.32, 0.45)	0.39 (0.32, 0.46)
Mogollon Rim and Mountains	0.06 (0.04, 0.09)	0.10 (0.07, 0.13)	0.10 (0.07, 0.13)	0.11 (0.07, 0.13)
Madrean Archipelago	0.02 (0.01, 0.03)	0.02 (0.01, 0.02)	0.02 (0.01, 0.02)	0.02 (0.01, 0.02)

Table S10. Mean predicted occupancy (2050-2059) and 95% credible intervals based on three future scenarios for each ecoregion for *Bombus occidentalis* in the western conterminous United States. Occupancy predictions generated from the refit model using USGS-CLC land cover.

	2050's Future Scenarios		
**Ecoregion**	**Best**	**Middle**	**Worst**
Blue Mountains	0.20 (0.16, 0.24)	0.04 (0.02, 0.07)	0.01 (0.00, 0.03)
Cascade Range	0.31 (0.26, 0.35)	0.08 (0.04, 0.11)	0.03 (0.01, 0.05)
Coastal Forest	0.23 (0.18, 0.26)	0.07 (0.04, 0.10)	0.03 (0.01, 0.04)
Colorado Plateau and Wyoming Basin	0.11 (0.08, 0.14)	0.03 (0.01, 0.04)	0.01 (0.00, 0.01)
Columbia Plateau	0.08 (0.05, 0.11)	0.03 (0.01, 0.05)	0.01 (0.00, 0.01)
Great Basin	0.10 (0.07, 0.14)	0.04 (0.01, 0.06)	0.01 (0.00, 0.02)
Greater Yellowstone Ecosystem	0.54 (0.48, 0.60)	0.24 (0.15, 0.31)	0.06 (0.02, 0.10)
Isolated Mtns in Prairie	0.25 (0.21, 0.29)	0.10 (0.06, 0.14)	0.03 (0.01, 0.06)
Mediterranean California	0.07 (0.04, 0.09)	0.02 (0.01, 0.02)	0.01 (0.00, 0.01)
Northern Rocky Mountains	0.35 (0.30, 0.39)	0.14 (0.09, 0.21)	0.03 (0.01, 0.05)
Southern Rocky Mountains	0.37 (0.32, 0.41)	0.11 (0.07, 0.15)	0.04 (0.01, 0.07)
Semi-arid Prairies	0.06 (0.04, 0.07)	0.03 (0.01, 0.03)	0.01 (0.00, 0.01)
Sierra Nevada	0.25 (0.20, 0.30)	0.08 (0.04, 0.11)	0.03 (0.01, 0.05)
Uinta and Wasatch Mtns	0.36 (0.29, 0.41)	0.16 (0.09, 0.22)	0.04 (0.01, 0.07)
Mogollon Rim and Mountains	0.18 (0.13, 0.23)	0.03 (0.01, 0.05)	0.01 (0.00, 0.02)
Madrean Archipelago	0.02 (0.01, 0.03)	0.01 (0.00, 0.01)	0.01 (0.00, 0.01)

Table S11. Percent change from occupancy estimated in 2020 to the mean of projected occupancy for 2050-2059 (2050's) by ecoregion for *Bombus occidentalis* in the western conterminous United States.

	2050's Future Scenarios		
**Ecoregion**	**Best**	**Middle**	**Worst**
Blue Mountains	-38 (-44, -32)	-87 (-92, -80)	-96 (-99, -91)
Cascade Range	18 (10, 27)	-72 (-81, -60)	-92 (-97, -83)
Coastal Forest	4 (-6, 15)	-70 (-81, -58)	-91 (-97, -82)
Colorado Plateau and Wyoming Basin	-2 (-11, 8)	-78 (-87, -68)	-96 (-99, -91)
Columbia Plateau	-49 (-55, -43)	-82 (-90, -73)	-97 (-99, -92)
Great Basin	-6 (-14, 3)	-72 (-83, -56)	-95 (-98, -87)
Greater Yellowstone Ecosystem	-21 (-24, -19)	-66 (-76, -56)	-93 (-97, -85)
Isolated Mtns in Prairie	-38 (-42, -33)	-76 (-83, -68)	-93 (-97, -86)
Mediterranean California	90 (36, 164)	-65 (-78, -48)	-87 (-96, -71)
Northern Rocky Mountains	-26 (-30, -22)	-70 (-80, -58)	-95 (-98, -89)
Southern Rocky Mountains	8 (-1, 17)	-69 (-78, -59)	-91 (-96, -81)
Semi-arid Prairies	-70 (-79, -58)	-89 (-95, -82)	-97 (-99, -94)
Sierra Nevada	66 (41, 95)	-51 (-66, -33)	-86 (-95, -70)
Uinta and Wasatch Mtns	-8 (-16, -2)	-62 (-74, -45)	-93 (-97, -85)
Mogollon Rim and Mountains	81 (52, 115)	-71 (-81, -59)	-92 (-97, -82)
Madrean Archipelago	52 (27, 84)	-88 (-93, -81)	-92 (-97, -83)

SI References

1. L. Richardson *et al.*, Bumble bee occurrences of North America from 1805–2020. *Dryad Digital Repository* (2002). https://doi.org/10.5061/dryad.c59zw3r8f.
2. T. A. Graves *et al.*, Bumblebees in Montana, North Dakota, South Dakota, and Nevada, USA, 2018-2022. U.S. Geological Survey data release. 2022. https://doi.org/10.5066/P931YWY8.
3. O. Lepais *et al.*, Estimation of bumblebee queen dispersal distances using sibship reconstruction method. *Mol. Ecol.* **19**, 819–831 (2010).
4. S. Jha, C. Kremen, Urban land use limits regional bumble bee gene flow. *Mol. Ecol.* **22**, 2483–2495 (2013).
5. M. M. Thornton *et al.*, Daymet: Daily Surface Weather Data on a 1-km Grid for North America, Version 4. ORNL DAAC, Oak Ridge, Tennessee, USA (2020). Available at: https://doi.org/10.3334/ORNLDAAC/1840. Accessed June 2022.
6. C. Daly *et al.*, Physiographically-sensitive mapping of temperature and precipitation across the conterminous United States. *Int. J. Climatol.* **28**, 2031–2064 (2008).
7. B. W. Allred *et al.*, Improving Landsat predictions of rangeland fractional cover with multitask learning and uncertainty. *Methods Ecol. Evol.* **12**, 841–849 (2021).
8. M. H. K. Hesselbarth, M. Sciaini, K. A. With, K. Wiegand, J. Nowosad, landscapemetrics: an open-source R tool to calculate landscape metrics. *Ecography* **42**, 1648–1657 (2019).
9. R Core Team, R: A language and environment for statistical computing. R Foundation for Statistical Computing, Vienna, Austria. (2021). Available at: https://www.R-project.org/.
10. USFWS, National Wetlands Inventory. U.S. Department of the Interior, Fish and Wildlife Service, Washington, D.C. (2018). Available at: https://www.fws.gov/program/national-wetlands-inventory/data-download. Accessed June 2022.
11. J. A. Royle, R. M. Dorazio, Hierarchical modeling and inference in ecology: the analysis of data from populations, metapopulations and communities. (Academic Press, 2008), pp. 1–26
12. T. A. Graves *et al.*, Western bumble bee: declines in the continental United States and range-wide information gaps. *Ecosphere* **11**, e03141 (2020).
13. B. Heinrich, Bumblebee economics. (Harvard University Press, 1979), pp. 51
14. D. Kenna, S. Pawar, R. J. Gill, Thermal flight performance reveals impact of warming on bumblebee foraging potential. *Funct. Ecol.* **35**, 2508–2522 (2021).
15. M. B. Hooten, N. T. Hobbs, A guide to Bayesian model selection for ecologists. *Ecol. Monogr.* **85**, 3–28 (2015).
16. A. Gelman, J. Hwang, A. Vehtari, Understanding predictive information criteria for Bayesian models. *Stat. Comput.* **24**, 997–1016 (2014).
17. S. Watanabe, Asymptotic equivalence of Bayes cross validation and widely applicable information criterion in singular learning theory. *J. Mach. Learn. Res.* **11**, 3571-3594 (2010).
18. W. M. Janousek, T. A. Graves, Western bumble bee predicted occupancy (1998, 2020) and future projection (2050s) rasters, western conterminous United States. U.S. Geological Survey data release. 2022. https://doi.org/10.5066/P9UHMCV1.

19. C. Hitaj *et al.*, Sowing uncertainty: what we do and don’t know about the planting of pesticide-treated seed. *BioScience* **70**, 390–403 (2020).

20. USDA National Agricultural Statistics Service Cropland Data Layer, National Cropland Data Layer 2008-2014. Available at: https://nassgeodata.gmu.edu/CropScape/. Accessed June 2021.

21. M. R. Douglas *et al.*, Putting pesticides on the map for pollinator research and conservation. *Sci. Data* **9**, 1–15 (2022).

22. W. M. Janousek, M. Douglas, T. A. Graves, Neonicotinoid nitroguanidine group insecticide application rates estimated across the western conterminous United States, 2008 to 2014. U.S. Geological Survey data release. 2022. https://doi.org/10.5066/P9H45NUG.

23. J. T. Abatzoglou, T. J. Brown, A comparison of statistical downscaling methods suited for wildfire applications. *Int. J. Climatol.* **32**, 772–780 (2012).

24. T. L. Sohl *et al.*, Spatially explicit modeling of 1992–2100 land cover and forest stand age for the conterminous United States. *Ecol. Appl.* **24**, 1015–1036 (2014).

25. T. L. Sohl *et al.*, Conterminous United States Land Cover Projections - 1992 to 2100: U.S. Geological Survey data release, (2018). Available at: https://doi.org/10.5066/P95AK9HP. Accessed June 2022.

26. K. C. Hegewisch, J. T. Abatzoglou, 'Future Climate Scatter' web tool. Climate Toolbox. Available at: https://climatetoolbox.org. Accessed on June 2022.

27. L. A. Joyce, D. Coulson, Climate scenarios and projections: A technical document supporting the USDA Forest Service 2020 RPA Assessment. Gen. Tech. Rep. RMRS-GTR-413. Fort Collins, CO: U.S. Department of Agriculture, Forest Service, Rocky Mountain Research Station. (2020), pp. 85
